# The Protective Paradox: Can School Connectedness Buffer Socioeconomic Disparities in Adolescent Mental Health?

**DOI:** 10.1002/jad.70157

**Published:** 2026-04-17

**Authors:** Esther Ariyo, Amurtiya Michael, Cindy Oreoluwa Ajala

**Affiliations:** ^1^ School of Health and Social Care University of Essex Essex UK; ^2^ Department of Agricultural Economics and Extension Modibbo Adama University Yola Nigeria; ^3^ Department of Sociology Faculty of Social science Osun State University Osogbo Nigeria

**Keywords:** adolescent mental health, LMICs, protective factors, resilience theory, school connectedness, socioeconomic status

## Abstract

**Introduction:**

Socioeconomic status (SES) is a well‐established factor influencing adolescents’ mental health, as young people from disadvantaged backgrounds are more likely to experience stress, anxiety, and poorer overall wellbeing. One factor that may help protect students from these negative outcomes is *school connectedness* which is the feeling of belonging, support, and positive relationships within the school environment. Although school connectedness is known to promote healthier psychological development, it remains unclear whether it can specifically reduce mental health inequalities linked to SES, especially in low‐ and middle‐income countries (LMICs).

**Methods:**

This study interrogates a critical gap in resilience theory: Can school connectedness effectively buffer the psychological consequences of socioeconomic disadvantage in resource‐constrained environments? Through a cross‐sectional design involving 535 Nigerian adolescents (mean age = 13.25 years; 53.6% female), we employed psychometrically validated measures including the California Healthy Kids Survey (school connectedness), WHO‐5 Wellbeing Index, and Kessler‐6 Psychological Distress Scale.

**Results:**

Result shows that school connectedness significantly predicted enhanced wellbeing (*β* = 1.98, *p *< 0.001) and reduced distress (*β* = −0.73, *p *= 0.001), but did not moderate the SES‐mental health relationship. This result challenges conventional assumptions in resilience frameworks and raises crucial questions about the contextual limits of school‐based protective factors in LMIC settings.

**Conclusions:**

The study makes three key contributions: (1) empirical evidence from an understudied Global South context, (2) theoretical contribution to the resilience models, and (3) practical implications for designing multi‐level mental health interventions in resource‐constrained educational systems.

## 1 Introduction

Socioeconomic status (SES) is a well‐established determinant of adolescent mental health. (Bradley and Corwyn 2002; Braveman et al. 2010; Marmot 2020; Reiss 2013). Adolescents from low‐SES backgrounds face higher risks for poor mental health, including low life satisfaction and increased psychological distress (Patel et al. 2007; World Health Organization 2021; Yoshikawa et al. 2012). School connectedness is students’ sense of being cared for, supported, and valued at school, is a recognized protective factor for adolescent mental health (Diggs et al. 2024; Goodenow 1993; McNeely et al. 2010; McNeely et al. 2002). We define school connectedness as a student's felt sense of belonging at school and of being known, respected, and supported by adults and peers (Marsh et al. 2019). In this study, we consider four interrelated components: (i) belonging and inclusion; (ii) caring adult–student relationships and fair, high expectations; (iii) positive, respectful peer relationships; and (iv) meaningful participation in a safe, orderly school climate (Wu et al. 2025). Public health agencies have highlighted school connectedness as a priority in youth suicide prevention, and experimental evidence shows benefits for behavioral outcome (Bonell et al. 2018; Shinde et al. 2018).

According to the Protective Factors Model of resilience theory, positive experiences like school connectedness can buffer the impact of risk (Masten and Cicchetti 2016; Zimmerman et al. 2013). Studies show its protective role against negative life events, including suicidal ideation and social deprivation (Goetschius et al. 2021; Gunn et al. 2018; Huang and Baxter 2021; Kim et al. 2020; Loukas and Pasch 2013). High connectedness is associated with lower distress, better emotional health, more prosocial behavior, and greater resilience (Kappi et al. 2023; Oldfield et al. 2016; Raniti et al. 2022; Resnick 1997; Sharp et al. 2018).

However, whether connectedness protects equally across socioeconomically diverse groups remains uncertain (Sampasa‐Kanyinga and Hamilton 2016). SES can shape opportunities to experience connectedness through school‐level structural conditions: under‐resourcing, large class sizes, limited pastoral support, staff turnover, and heavy teacher workload can constrain day‐to‐day interactions that build trust and belonging. These structural features, alongside out‐of‐school constraints associated with poverty (e.g., time pressure, transport costs, caregiving responsibilities), may limit access to the very relational supports that confer protection (Bonny et al. 2000; Fletcher and Bonell 2013; Markham et al. 2021; Moore et al. 2017; Shackleton et al. 2018). As such, the very adolescents who stand to benefit most from school connectedness may struggle to access it due to structural inequalities. Most studies examine SES and connectedness as separate predictors or treat SES primarily as a determinant of connectedness, overlooking the dynamic interplay between structural disadvantage and relational supports. A key question arises: Can school connectedness still serve as a protective factor for adolescents who face barriers to accessing it? This study examines links between adolescent well‐being, distress, and school connectedness in Nigeria, focusing on its moderating role between SES and mental health.

### 1.1 Current Study

This study advances understanding of how protective factors function under risk, an essential concern in resilience theory and youth development models (Fergus and Zimmerman 2005). In LMICs where formal mental health services are limited, identifying accessible supports in adolescents’ everyday settings is critical. This aligns with ecological systems and resilience frameworks, which emphasize the role of immediate social contexts like schools in shaping responses to adversity (Bronfenbrenner and Morris 2006; Masten and Cicchetti 2016).

Mental health in adolescence is complex. While research has traditionally focused on psychological distress (e.g., depression, anxiety), there is growing recognition of the need to assess positive mental health indicators like subjective well‐being (Huppert and So 2013; Keyes 2005). A narrow focus on distress may miss youth who are not clinically ill but lack psychological flourishing. A balanced approach considers both well‐being and distress (World Health Organization 2021).

Most evidence on school connectedness and mental health comes from high‐income countries, where cultural and systemic differences may limit generalizability to LMICs (Motti‐Stefanidi and Masten 2017; OECD 2013). Cultural contexts shape how school connectedness impacts well‐being (García‐Moya et al. 2019). A meta‐analysis by Yuen and Wu (2024) showed variations across individualistic and collectivist societies. We also consider conceptual equivalence. These differences highlight the need to consider whether the construct retains conceptual equivalence in non‐Western contexts. We treat school connectedness as a social–ecological construct with a common core across cultures but contextually shaped expressions. For example, in many non‐Western settings, respect for adults, communal obligations, and perceptions of safety and fairness may be especially salient, whereas in other settings student voice and extracurricular participation may be more central. We adopt the definition above and interpret findings with these cultural contingencies in view, situating results within the Nigerian school context.

In the Nigerian school context, school connectedness may be expressed through culturally specific pathways. Respectful and supportive teacher–student relationships are central, reflecting broader social norms that emphasize deference to adults and moral guidance from authority figures (Olawale Olatunji 2025; Omodan and Tsotetsi 2018). Connectedness is also shaped by communal forms of belonging, including shared rituals such as morning assemblies, collective responsibilities, and house‐based group activities that promote solidarity (Teklemariam 2025). Peer connectedness often reflects strong group identity and interdependence rather than individual autonomy. A sense of safety, fairness, and emotional support from teachers who frequently act as mentors beyond academic instruction is especially salient in settings where discipline practices, class sizes, and resource constraints vary widely. These pathways highlight that while the core construct of school connectedness may be universal, its lived expression is contextually shaped in Nigeria.

In Nigeria, where schools may be one of the few consistent support structures, understanding these dynamics is particularly urgent especially for youth facing poverty or instability. Additionally, as global inequality grows, understanding how school connectedness works for students of diverse SES becomes increasingly vital to developing appropriate interventions for adolescents (Dennis et al. 2022; Nolan et al. 2019).

We address these key gaps in the literature by examining both positive indicators (subjective well‐being) and negative indicators (psychological distress) in relation to school connectedness. Our guiding research questions are:

1. Is school connectedness associated with subjective well‐being and psychological distress?
2. Does school connectedness moderate the relationship between socioeconomic disadvantage and adolescent subjective well‐being and psychological distress?

The study contributes to resilience theory by testing whether school connectedness a modifiable, relational factor buffers the impact of socioeconomic risk (Fergus and Zimmerman 2005; Masten 2014). The resilience model specifically suggests that when adolescents face elevated risk, such as poverty, protective factors, like supportive relational factors like school environments, can moderate or “buffer” the impact of those risks on outcomes such as mental health. Positioned as a social‐ecological resource, school connectedness may mitigate structural disadvantage. Understanding when and for whom it is effective enhances insight into how protective factors operate under adversity, particularly in under‐resourced settings.

By offering context‐specific evidence from Nigeria, this research addresses a critical gap in cross‐cultural psychology and global adolescent mental health. This study supports the design of equitable, scalable school‐based interventions in LMICs (Motti‐Stefanidi and Masten 2017; Unicef Innocenti Research Centre 2020). It will strengthen the evidence base for using school connectedness to promote resilience and well‐being among adolescents facing socioeconomic adversity.

## 2 Methods

### 2.1 Data Collection

Nigeria, Africa's most populous country, follows a 6‐3‐3‐4 education system: 6 years of primary, 6 years of secondary (split into junior and senior secondary, typically for ages 12–18), and 4 years of tertiary education. English is the official language of instruction. Public schools, which serve most students especially in low‐income and rural areas are government‐funded. In contrast, private schools, often better resourced, cater to middle‐ and high‐income families. Secondary schools in Nigeria tend to be socioeconomically homogenous.

Osun State, in Nigeria's southwest, includes both urban and semi‐urban areas and offers a mix of public and private secondary schools. Schools in Osun, like many parts of Nigeria, face challenges such as poverty and educational inequality, making it a relevant setting to examine how school connectedness and SES impact adolescent mental health in low‐resource contexts.

Data were collected from 680 students across 5 randomly selected secondary schools (public and private) in Okuku and Osogbo, Osun State. A pre‐test with 30 students in Ogun State ensured the survey tools were clear and feasible. Within each school, students were randomly selected from all class levels. Participation was voluntary, with informed consent obtained from both students and their teachers.

Questionnaires were administered in classrooms during school hours, with teachers present only to maintain order. The process maintained anonymity and confidentiality. Ethical approval was granted by the Ogun Health Research Ethics Committee (OGHREC/467/06) and all procedures followed standard guidelines for research with human participants.

### 2.2 Measures

#### 2.2.1 School Connectedness (Independent Variable)

School connectedness was assessed using the 5‐item California Healthy Kids Survey School Connectedness Scale (WestEd 2021). Items were rated on a 5‐point Likert scale (1 = strongly disagree to 5 = strongly agree), with higher scores indicating stronger connectedness. The average of the five items was used, showing good internal consistency in this study (*α *= 0.84). This scale has demonstrated strong reliability and validity among adolescents, including Black populations (Furlong et al. 2011).

#### 2.2.2 Subjective Well‐Being (Dependent Variable)

Subjective well‐being was measured using the WHO‐5 Well‐Being Index (World Health Organization 1998). Students rated how often they experienced positive feelings over the past 2 weeks on a 6‐point scale (0 = at no time to 5 = all of the time). Total scores range from 0 to 25, with higher scores indicating better well‐being. The scale showed high reliability (*α* = 0.86) and is validated for adolescents in LMICs (Topp et al. 2015).

#### 2.2.3 Psychological Distress (Dependent Variable)

The Kessler‐6 (K6) scale (Kessler et al. 2002), assessed psychological distress through six items on symptoms like nervousness and hopelessness over the past 30 days. Responses were rated on a 5‐point scale (0 = none of the time to 4 = all of the time), with scores ranging from 0 to 24. Scores ≥ 13 indicate serious distress. The K6 had good reliability in this study (*α* = 0.75).

#### 2.2.4 Control Variables

Demographics included age, sex (1 = male, 2 = female), and school type (1 = private, 2 = public).

#### 2.2.5 SES Measure

Two SES indicators were selected including parental education and household asset index following established LMIC methodologies (Filmer et al. 2001; Howe et al. 2009). This approach addresses the unreliability of adolescent self‐reported income data (Currie et al. 2008) and social comparison biases (Goodman et al. 2001), while capturing both material and social dimensions of disadvantage (Ensminger and Fothergill 2014).

Parental education was calculated as the average of the mother's and father's education, each coded from 1 (*no education*) to 4 (*tertiary education*), with higher scores reflecting greater educational attainment.

Household asset‐based SES index. A Principal Component Analysis (PCA) was conducted on 10 household asset items to construct a socioeconomic status (SES) index and a food insecurity question. Variables for household ownership includes questions on flooring quality, access to water within the house, shared toilet facility with neighbors, use of gas as cooking fuel, school books possession, at least five good clothes possession, access to internet, sleeping material, ownership of personal table and ownership of phone). Food insecurity was assessed with the item, “How often have you been hungry in the last 30 days because there was no food?”, rated on a 5‐point scale (1 = *never* to 5 = *always*). It was recoded to a binary variable for never as “1” and 2 to 5 as “0”. Two Items (sharing of toilet and use of gas for cooking) were excluded due to low communalities or weak loading. The final SES index includes 9 binary items, which explained 22.89% of the total variance and was retained as a continuous SES measure with higher values indicating higher socioeconomic status. This approach is consistent with prior methods used in the DHS and similar socioeconomic research studies (Filmer et al. 2001; Rutstein 2004). The KMO measure was 0.69, indicating sampling adequacy, and Bartlett's Test of Sphericity was significant (*χ*²(36) = 385.644, *p *< 0.001), supporting the use of PCA. The SES household index used in this study includes indicators that reflect both the adolescent's status (e.g., personal items) and general household conditions, providing a more comprehensive picture of living standards.

### 2.3 Data Analysis

**Figure 1:**
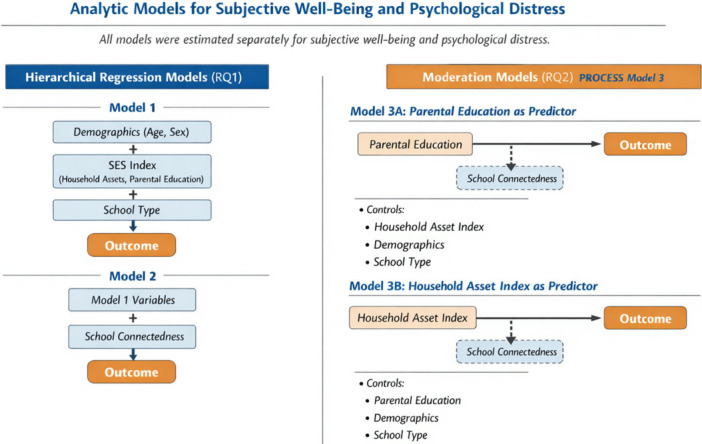
Tested regression models.

Three hierarchical regression models were run. Model 1 included demographics (sex, age), SES (household assets, parental education), and school type. Model 2 added school connectedness. Model 3 tested moderation using the PROCESS macro for SPSS (Hayes 2017), with school connectedness as the moderator and SES indicators as predictors based on their significance in prior models. To address Research Question 2, moderation analyses were conducted using PROCESS. Two models were tested: Model 3A, with parental education as the predictor and SES controlled; and Model 3B, with SES as the predictor and parental education controlled. This approach acknowledges the multidimensional nature of SES and isolates the unique effect of each indicator. Figure 1 provide a summary of the model tested. Analyses were conducted separately for subjective well‐being and psychological distress.

Regression assumptions were met. Residual plots and histograms showed no major violations of linearity, normality, or homoscedasticity. Durbin‐Watson values (1.61 and 1.86) indicated independent errors. No multicollinearity concerns were found (VIFs < 2.1; tolerance > 0.50). Continuous predictors were mean‐centered before creating interaction terms. Though Breusch‐Pagan tests indicated heteroscedasticity, PROCESS used robust standard errors, ensuring valid results. No significant outliers or influential cases were identified.

Descriptive statistics and bivariate correlations were also performed. Missing data were under 3% for most variables, except parental education (18%), likely reflecting students’ limited knowledge of this information. Little's MCAR test was significant (*χ*²(34) = 68.90, *p*< 0.001), indicating that the data were not MCAR. Because PROCESS does not support multiple imputation, analyses used listwise deletion (*n *= 535). To evaluate potential bias, we compared included and excluded students and modeled missingness via logistic regression (Tables 1 and 2). Missingness was mainly associated with younger age and private school type, whereas wellbeing, distress, and SES were not related to missingness. These predictors were included as covariates in all primary analyses, supporting the conclusion that listwise deletion is unlikely to have materially biased the results (Enders 2022; Graham 2009). Analyses were conducted using IBM SPSS Statistics (Version 29).

**Table 1:** Comparison of included and excluded participants based on parental education missingness.

Variable	Included (*n *= 551–558) *M* (SD)	Excluded (*n* = 121–122) *M* (SD)	*t* (df)	*p*	Effect size
Age (years)	13.40 (1.92)	12.55 (1.97)	4.43 (670)	< 0.001	*d* = 0.45
Psychological distress	5.19 (3.64)	5.42 (3.86)	−0.61 (668)	0.54	*d* = −0.06
Subjective wellbeing	14.73 (7.21)	14.12 (6.90)	0.85 (677)	0.40	*d* = 0.09
School connectedness	4.19 (0.74)	3.99 (0.72)	2.75 (678)	0.006	*d* = 0.28

*Note:* Independent‐samples *t* tests were conducted assuming equal variances (Levene's tests ns). Cohen's *d* values of 0.20, 0.50, and 0.80 correspond to small, medium, and large effects, respectively. Sample sizes vary slightly due to variable‐level missingness.

**Table 2:** Logistic regression predicting missing parental education (1 = Missing, 0 = Observed).

Predictor	*B*	SE	Wald *χ*²	*p*	Odds ratio (Exp(*B*))
Psychological distress	0.00	0.03	0.00	0.997	1.00
Household asset index	0.03	0.11	0.05	0.818	1.03
Subjective wellbeing	−0.03	0.02	3.54	0.060	0.97
School connectedness	−0.24	0.15	2.64	0.104	0.79
Age	−0.20	0.06	11.15	< 0.001	0.82
Sex	0.01	0.22	0.00	0.974	1.01
Type of school (public vs. private)	−1.25	0.42	8.85	0.003	0.29
Constant	3.97	1.12	12.57	< 0.001	52.70

*Note:* The model was statistically significant, *χ*²(7) = 37.64, *p* < 0.001, explaining between 5.7% (Cox & Snell *R*²) and 9.3% (Nagelkerke *R*²) of the variance in missing parental education. Odds ratios below 1 indicate lower odds of missing parental education.

## 3 Result

### 3.1 Descriptive Result

Descriptive statistics and correlations are shown in Tables 3 and 4. Participants averaged 13 years of age and were predominantly female. Most attended private schools. SES index was standardized (*M* = 0, SD = 1). Scores ranged from −2.45 to 1.70 with a slight negative skew (–0.36) and kurtosis of –0.76, indicating a fairly symmetrical distribution. Higher values reflected higher SES. Overall, students reported high levels of school connectedness and moderate subjective well‐being and psychological distress (Table 3).

**Table 3:** Descriptive statistics for study variables.

Continuous variables	*N*	*M*	SD
Age	672	13.25	1.96
parental education	558	3.62	0.69
Psychological distress	670	5.23	3.68
Subjective wellbeing	679	14.62	7.16
School connectedness	680	4.16	0.74
SES index	667	0.00	1.00

Categorical variables	Category	*N*	%
Sex	Male	314	46.4
	Female	363	53.6
Type of school	Private	536	79.3
	Public	140	20.7

*Note:* SES index is standardized (*M* = 0, SD = 1). Higher values reflect higher SES. School connectedness measured on a 5‐point scale.

**Table 4:** Correlations among study variables.

Variable	1	2	3	4	5	6	7	8
1. Sex	—							
2. Age	0.03	—						
3. School type	0.16**	0.29**	—					
4. Household asset index	−0.14**	0.12**	−0.17**	—				
5. Psychological distress	−0.02	0.12**	−0.06	−0.10**	—			
6. Parental education	−0.13**	−0.22**	−0.63**	0.15**	−0.02	—		
7. Subjective well‐being	−0.03	−0.10**	−0.30**	0.16**	−0.20**	0.29**	—	
8. School connectedness	0.07	−0.05	0.26**	−0.00	−0.19**	−0.14**	0.11**	—

*Note:* Values are Pearson correlation coefficients (*r*). Numbers ranged from 548 to 680 due to missing data.

** *p *< 0.01 (two‐tailed).

### 3.2 Differences in School Connectedness by School Type

We examined differences in school connectedness by school type because school type represents a key structural factor in the educational context and was meaningfully associated with the outcomes in preliminary analyses. Tests of normality indicated that connectedness scores were non‐normally distributed in both groups private schools (*n* = 536) Shapiro–Wilk *W *= 0.922, *p* < 0.001; public schools (*n* = 140) *W* = 0.831, *p* < 0.001 (Kolmogorov–Smirnov tests were also significant, both *p* < 0.001). Given the large sample sizes and the robustness of Welch's *t*‐test to violations of normality and unequal variances, we used it as the primary comparison and complemented it with a Mann–Whitney *U* test as a nonparametric sensitivity check. Both analyses showed that public‐school students reported significantly higher school connectedness than private‐school students, with Welch's test indicating a moderate‐to‐large effect size and the Mann–Whitney test yielding the same directional pattern. These findings confirm a substantive difference in connectedness between school sectors. Details are in Table 5.

**Table 5:** Differences in school connectedness by type of school.

Type of school	*n*	*M*	SD
Private	536	4.06	0.76
Public	140	4.53	0.51

Inferential statistics				
Test	Statistic	*df*	*p*	Effect size
Welch's *t* test	*t* = −8.67	316.69	< 0.001	*d* = −0.66
Mann–Whitney *U* (sensitivity)	*U* = 22,752	—	< 0.001	*r* = 0.28^†^

*Note:* A Mann–Whitney *U* test was conducted as a sensitivity analysis because school connectedness was not normally distributed. School type coded as 1 = private, 2 = public.^†^Effect size r for the Mann–Whitney test was calculated as Z/√N.Cohen's d is reported for the *t* test.

Abbreviations: *M*, mean; SD, standard deviation.

### 3.3 Regression Result

#### 3.3.1 Subjective Well‐Being

As shown in Table 6, Model 1 explained 13.1% of the variance in subjective well‐being (*R*² = 0.131, F(5, 529) = 15.94, *p *< 0.001). Parental education (*b* = 1.56, SE = 0.57, *p *= 0.007), SES asset index (*b* = 0.86, SE = 0.30, *p *= 0.004), and school type (*b* = −3.22, SE = 0.93, *p *< 0.001) were significant predictors. This suggests that adolescents with more educated parents, higher SES, and those attending private schools reported greater well‐being. Sex and age were not significant.

**Table 6:** Hierarchical multiple regression predicting subjective wellbeing (*N* = 535).

Predictor variable	Model 1B (SE)	*β*	Model 2B (SE)	*β*	Model 3A: parental education × SC B (SE)	Model 3B: SES × SC B (SE)
Constant	15.46 (3.67)***	—	6.46 (4.06)	—	20.47 (2.38)***	15.20 (3.62)***
Sex	0.19 (0.60)	0.01	0.21 (0.59)	0.02	0.20 (0.59)	0.24 (0.59)
Age	−0.19 (0.16)	−0.05	−0.06 (0.16)	−0.02	−0.06 (0.16)	−0.08 (0.16)
School type	−3.22 (0.93)***	−0.19	−4.24 (0.94)***	−0.25	−4.21 (0.95)***	−4.36 (0.94)***
Household asset index (SES)	0.86 (0.30)**	0.12	0.78 (0.29)**	0.11	0.78 (0.29)**	0.78 (0.29)**
Parental education	1.56 (0.57)**	0.15	1.59 (0.56)**	0.15	1.57 (0.57)**	1.56 (0.56)**
School connectedness	—	—	1.98 (0.42)***	0.20	1.96 (0.44)***	2.03 (0.42)***
Interaction term	—	—	—	—	0.15 (0.79)	−0.41 (0.37)
*R*²	0.131		0.167		0.167	0.169
Δ*R*²	—		0.036***		0.000	0.002
Adjusted *R*²	0.123		0.157		0.156	0.158
*F* for model	15.94***		17.63***		15.09***	15.28***

*Note:* Standardized coefficients (*β*) are reported for hierarchical regression models (Models 1 and 2). Moderation analyses (Models 3A and 3B) were conducted using the PROCESS macro for SPSS, which reports unstandardized coefficients for interaction models.

Abbreviations: Δ*R*², *R*² change; *B*, unstandardized regression coefficient; SC, school connectedness; SE, standard error of *B*.

** *p* < 0.01.

*** *p *< 0.001.

In Model 2, the addition of school connectedness significantly improved the model, explaining an additional 3.6% of the variance (Δ*R*² = 0.036, *F* change = 22.78, *p *< 0.001). School connectedness was a strong positive predictor (*b* = 1.98, SE = 0.42, *p *< 0.001). Parental education (*b* = 1.59, SE = 0.56, *p *= 0.005), SES (*b* = 0.78, SE = 0.29, *p *= 0.008), and school type (*b *= −4.24, SE = 0.94, *p *< 0.001) remained significant. These results support Research Question 1, confirming that school connectedness is a robust independent predictor of adolescent well‐being.

In Model 3A, the interaction between parental education and school connectedness was not significant (*b* = 0.15, SE = 0.79, *p *= 0.847). However, both parental education (*b* = 1.57, SE = 0.57, *p *= 0.006) and school connectedness (*b* = 1.96, SE = 0.44, *p *< 0.001) remained significant predictors. The SES asset index also remained significant (*b* = 0.78, SE = 0.29, *p *= 0.008). The model explained 16.7% of the variance (*R*² = 0.167).

In Model 3B, the interaction between SES and school connectedness was also not significant (*b *= –0.41, SE = 0.37, *p *= 0.278). However, school connectedness (*b* = 2.03, SE = 0.42, *p *< 0.001), SES (*b* = 0.78, SE = 0.29, *p *= 0.008), and parental education (*b* = 1.56, SE = 0.56, *p *= 0.005) all remained significant. The model explained 16.9% of the variance (*R*² = 0.169).

**Figure 2:**
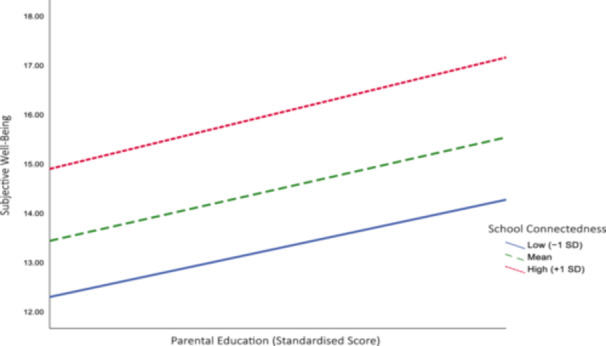
Association between parental education and subjective well‐being at low (−1 SD), mean, and high (+1 SD) levels of school connectedness. Predicted values are based on PROCESS Model 3A controlling for sex, age, school type, and Household Asset Index.

**Figure 3:**
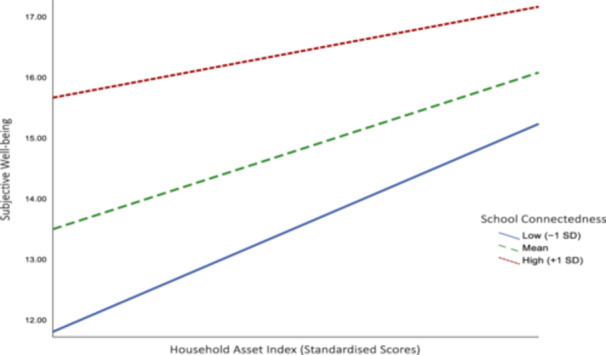
Association between parental education and subjective well‐being at low (−1 SD), mean, and high (+1 SD) levels of school connectedness. Predicted values are based on PROCESS Model 3B controlling for sex, age, school type, and parental education.

These findings indicate that while school connectedness is a strong independent predictor of subjective well‐being, it does not moderate the relationship between SES and well‐being. See Figures 2 and 3.

#### 3.3.2 Psychological Distress

**Figure 4:**
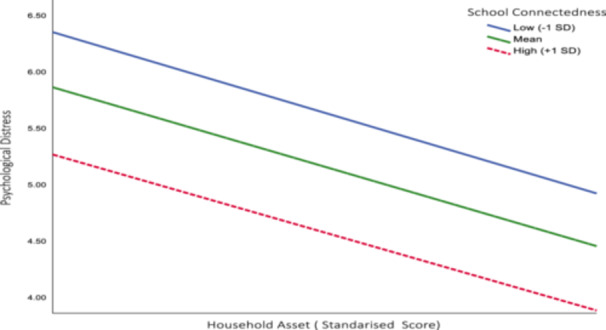
Association between parental education and psychological distress at low (−1 SD), mean, and high (+1 SD) levels of school connectedness. Predicted values are based on PROCESS Model 3A controlling for sex, age, school type, and Household Asset Index.

Results for psychological distress are presented in Table 7. In Model 1, SES (*b* = –0.45, *p *= 0.005) was a significant predictor of psychological distress, while type of school (*b *= –0.81, *p *= 0.107) and parental education (*b *= –0.10, *p *= 0.739) were not. The model explained 4.2% of the variance in distress (*R*² = 0.042, *p *< 0.001), indicating that adolescents with lower SES reported higher levels of psychological distress. Model 2 included school connectedness, which significantly improved the model by accounting for an additional 1.9% of the variance (Δ*R*² = 0.019, *p *= 0.001). School connectedness was a significant negative predictor (*b *= –0.73, *p *= 0.001), suggesting that adolescents who felt more connected at school reported lower psychological distress. SES remained significant (*b *= –0.42, *p *= 0.008), while parental education remained non‐significant. In Model 3, the interaction between school connectedness and SES was not significant (*b* = 0.01, *p *= 0.961), indicating no moderating effect. However, school connectedness (*b *= –0.73, *p *= 0.001) and SES (*b *= –0.42, *p *= 0.008) remained significant predictors, highlighting their independent contributions to lower distress levels. See Figure 4.

**Table 7:** Hierarchical multiple regression predicting psychological distress (*N* = 535).

Predictor	Model 1B (SE)	Model 1β	Model 2B (SE)	Model 2β	Model 3B (SE)
Constant	3.27 (1.95)	—	6.55 (2.18)**	—	3.49 (1.94)
Sex	−0.60 (0.32)†	−0.08	−0.62 (0.32)†	−0.08	−0.62 (0.32)†
Age	0.31 (0.09)***	0.17	0.26 (0.09)**	0.14	0.26 (0.09)**
School type	−0.81 (0.50)	−0.10	−0.42 (0.51)	−0.05	−0.42 (0.51)
Household asset index	−0.45 (0.16)**	−0.13	−0.42 (0.16)**	−0.12	−0.42 (0.16)**
Parental education	−0.10 (0.31)	−0.02	−0.11 (0.30)	−0.02	−0.10 (0.30)
School connectedness	—	—	−0.73 (0.22)**	−0.15	−0.73 (0.23)**
SES × School connectedness	—	—	—	—	0.01 (0.20)
*R*²	0.042		0.061		0.061
Δ*R*²	—		0.019**		0.000
*F* for Model	4.63***		5.70***		4.88***
Adjusted *R*²	0.033		0.050		0.049

*Note:* Standardized coefficients (*β*) are reported for hierarchical regression models (Models 1 and 2). Moderation analyses (Model 3A) was conducted using the PROCESS macro for SPSS, which reports unstandardized coefficients for interaction models.

Abbreviations: Δ*R*², *R*² change; *B*, unstandardized regression coefficient; SE, standard error of *B*.

^†^ *p *< 0.10.

** *p* < 0.01.

*** *p* < 0.001.

## 4 Discussion

This study examined the role of school connectedness in adolescent mental health within the Nigerian context, focusing on its association with subjective well‐being and psychological distress, and its potential to buffer the negative effects of socioeconomic disadvantage. The findings contribute to a growing body of literature that positions social relationships within the school environment as central to adolescent development, while offering new insights from a low‐ and middle‐income country (LMIC) perspective.

Consistent with global research, school connectedness emerged as a key predictor of better mental health outcomes. Evidence from high‐income countries has shown that adolescents who feel supported, accepted, and included at school report greater well‐being and fewer symptoms of distress (Bond et al. 2007; Jose et al. 2012). This study extends that evidence to Nigeria, highlighting that the mental health benefits of school connectedness may operate across diverse cultural and economic contexts. These findings suggest a level of universality in the protective value of school belonging, even in under‐resourced settings.

However, school connectedness did not moderate the negative relationship between adolescent mental health and SES. Given that interaction effects typically require larger samples and full variability to detect, especially when SES ranges are narrow, the absence of moderation should be interpreted cautiously. Future research using larger samples is needed to more conclusively determine whether moderation effects exist. Importantly, there were also meaningful differences by school type. Public‐school students reported stronger connections to their schools than their private‐school peers, yet their overall well‐being was lower when broader contextual factors were considered. This suggests that while public schools may foster stronger relationships, wider pressures such as resource limitations, classroom conditions, environmental stress, or economic hardship may dampen well‐being despite high levels of connectedness. In this context, connectedness remains beneficial but cannot, on its own, overcome broader systemic constraints. This finding contributes to ongoing efforts to refine resilience theory by showing that protective factors like school connectedness may have universal effects but are not always sufficient to offset the effects of entrenched structural risks in resource‐limited environments (Masten 2018; Ungar 2019).

This suggests that in LMICs, where poverty and under‐resourced school systems are widespread, even strong school relationships may not fully compensate for economic hardship. This emphasizes the need for multi‐layered resilience strategies that combine relational supports (like school connectedness) with broader structural and policy‐level interventions (Masten 2018; Ungar 2019). Practical strategies for enhancing connectedness in resource‐constrained contexts include fostering respectful and supportive teacher–student relationships, promoting inclusive classroom practices, and encouraging peer support and student participation. When embedded within broader efforts to improve school conditions and social protection, these relational practices may help buffer everyday stressors and support emotional safety. Importantly, they rely on social and organizational processes rather than material resources, making them feasible within existing school infrastructures.

School connectedness is therefore a realistic focus for mental health promotion, particularly in low‐resource settings. Because it is shaped by everyday interactions and school culture, it is considered a modifiable factor that can be strengthened without requiring large financial investments. This makes it a low‐cost and scalable intervention point (Allen et al. 2018; Barry et al. 2017; Bonell et al. 2018; Shinde et al. 2018). In contexts where mental health services are scarce, such as Nigeria and many LMICs, schools represent one of the few institutions that reach most adolescents (Motti‐Stefanidi and Masten 2017; Shinde et al. 2018; Unicef Innocenti Research Centre (2020). Importantly, strengthening school connectedness should be viewed as complementary to, rather than a substitute for, broader policy and structural interventions aimed at reducing socioeconomic inequality.

However, some limitations must be acknowledged. First, the cross‐sectional design limits causal interpretations. Longitudinal studies are needed to track the temporal sequence between school connectedness and mental health outcomes (Gunn et al. 2018), particularly to understand how protective factors operate across developmental periods (Masten and Cicchetti 2016). Existing multi‐wave designs demonstrate predictive validity over years (Bond et al. 2007), but LMIC‐specific evidence remains limited. Second, reliance on adolescent self‐reports may introduce bias, especially regarding sensitive measures like distress. Third, although the SES index followed established asset‐based methodologies (Howe et al. 2009; Rutstein 2004), recent critiques highlight its inability to capture dynamic economic mobility (Hruschka et al. 2015), or multidimensional deprivations (Alkire and Santos 2014).

Future research should adopt longitudinal and mixed‐method designs to examine how school connectedness evolves over time and interacts with shifting economic conditions. More work is also needed to explore the role of teacher practices, peer dynamics, and school leadership styles in shaping connectedness within under‐resourced environments. Cross‐cultural comparative studies between LMICs and high‐income countries may further illuminate context‐specific dynamics and inform global adaptation of school‐based interventions.

Policy‐wise, integrating school connectedness into educational and mental health strategies could support adolescent well‐being. This includes embedding social‐emotional learning into curricula, training teachers to foster inclusive classroom climates, and improving school infrastructure particularly in public schools. However, broader structural reforms addressing poverty and educational inequality remain essential for sustainable improvements.

Clinicians and school‐based mental health practitioners should recognize school connectedness as a protective factor and incorporate it into prevention and early intervention strategies. Interventions should be tailored to socioeconomically disadvantaged youth who may face barriers to accessing relational support. Clinicians should routinely explore the quality of school relationships when evaluating adolescent well‐being.

This study highlights school connectedness as a robust and modifiable contributor to adolescent mental health in Nigeria. Although it does not weaken the effects of socioeconomic disadvantage, it consistently supports better well‐being across groups. Differences across school types emphasize the importance of considering school environments alongside broader structural conditions. Strengthening everyday relational practices together with wider investments in school resources and social protection offers a promising, multi‐layered approach to promoting adolescent well‐being in LMICs.

Conclusively, while school connectedness alone cannot eliminate the mental health risks associated with poverty, it remains a vital, scalable lever for promoting resilience and well‐being, particularly when embedded within broader structural efforts aimed at reducing socioeconomic disparities and strengthening adolescent support systems.

## Author Contributions

**Esther Ariyo:** conceptualization, methodology, writing – review and editing, supervision, writing – original draft, formal analysis, visualization. **Amurtiya Michael:** Resources, data curation, writing – review and editing. **Cindy Oreoluwa Ajala:** data curation, resources, investigation.

## Funding

The authors have nothing to report.

## Ethics Statement

Ethical approval for the study was obtained from the Ogun Health Research Ethics Committee (Ref: NHREC/25/01/OGHSHREC/23A). Written informed consent obtained from both students and their teachers.

## Conflicts of Interest

The authors declare no conflicts of interest.

## Data Availability

The data that support the findings of this study are available on request from the corresponding author. The data are not publicly available due to privacy or ethical restrictions.
